# Risk Factors and Outcomes for Postoperative Delirium after Major Surgery in Elderly Patients

**DOI:** 10.1371/journal.pone.0136071

**Published:** 2015-08-20

**Authors:** Jelle W. Raats, Wilbert A. van Eijsden, Rogier M. P. H. Crolla, Ewout W. Steyerberg, Lijckle van der Laan

**Affiliations:** 1 Department of Surgery, Amphia Hospital, Breda, The Netherlands; 2 Department of Public Health, Erasmus MC, Rotterdam, The Netherlands; University of Brescia, ITALY

## Abstract

**Background:**

Early identification of patients at risk for delirium is important, since adequate well timed interventions could prevent occurrence of delirium and related detrimental outcomes. The aim of this study is to evaluate prognostic factors for delirium, including factors describing frailty, in elderly patients undergoing major surgery.

**Methods:**

We included patients of 65 years and older, who underwent elective surgery from March 2013 to November 2014. Patients had surgery for Abdominal Aortic Aneurysm (AAA) or colorectal cancer. Delirium was scored prospectively using the Delirium Observation Screening Scale. Pre- and peri-operative predictors of delirium were analyzed using regression analysis. Outcomes after delirium included adverse events, length of hospital stay, discharge destination and mortality.

**Results:**

We included 232 patients. 51 (22%) underwent surgery for AAA and 181 (78%) for colorectal cancer. Postoperative delirium occurred in 35 patients (15%).

Predictors of postoperative delirium included: delirium in medical history (Odds Ratio 12 [95% Confidence Interval 2.7–50]), advancing age (Odds Ratio 2.0 [95% Confidence Interval 1.1–3.8]) per 10 years, and ASA-score ≥3 (Odds Ratio 2.6 [95% Confidence Interval 1.1–5.9]). Occurrence of delirium was related to an increase in adverse events, length of hospital stay and mortality.

**Conclusion:**

Postoperative delirium is a frequent complication after major surgery in elderly patients and is related to an increase in adverse events, length of hospital stay, and mortality. A delirium in the medical history, advanced age, and ASA-score may assist in defining patients at increased risk for delirium. Further attention to prevention of delirium is essential in elderly patients undergoing major surgery.

## Introduction

The number of people over 65 years is increasing and will continue to do so over the coming decades. Similarly, the number of elderly patients requiring surgery is expected to increase. Delirium is a common and serious problem in hospitalized patients, especially in the elderly. Postoperative delirium is associated with an increase in postoperative complications, a decrease in functional capacity, a prolonged hospital stay and a direct increase of healthcare costs [1–6].

Early identification of patients at risk for delirium is important because adequate well timed interventions could prevent occurrence of delirium and the related detrimental outcome.

Several prediction models have been developed, including multiple risk factors for postoperative delirium [7–9]. However, these studies are of varying quality and each with a heterogeneous population.

Measuring frailty may be a more sensitive marker of determining post-operative delirium [10]. However, to this date, there is no consensus on a clear definition and quantification of frailty. Several assessment instruments have been developed for frailty during the last decades. The most evidence based process to identify frail patients at this moment is comprehensive geriatric assessment. However, this is a resource intensive, time consuming process and therefore not suitable for clinical practice [11,12].

Preventing delirium is probably most effective in elective surgery because preventive actions could be initiated timely. Aortic Abdominal Aneurysm (AAA) and colorectal surgery are among the most performed elective major interventions and are hence of interest to study in detail. The primary objective of this study was to evaluate predictors of delirium, including factors describing frailty, in elderly patients undergoing elective colorectal or AAA surgery. Secondary outcome measures were the clinical consequences of delirium including adverse events, length of stay and mortality.

## Methods

### Patient selection

We prospectively registered data on patients of 65 years and older, who underwent surgery from March 2013 to November 2014. All patients underwent surgery in an elective setting at the Amphia Hospital, Breda, the Netherlands. We included patients having surgery for AAA and colorectal cancer. Exclusion criteria were: patients who were discharged within 2 days, patients receiving non-operative treatment, and patients who underwent non-elective (emergency) surgery. Emergency surgery included ruptured or symptomatic AAA surgery, or colorectal surgery with pre-operative obstructive ileus, active bleeding from colorectal cancer resulting in hemodynamic instability or perforation of bowel. The medical ethical committee of the Amphia Hospital in Breda, the Netherlands, permitted this project and waived informed consent.

### Delirium

Delirium was scored prospectively using the Delirium Observation Screening Scale (DOSS) [13,14]. The scale used was a shortened version with 13 items and was scored three times a day by a nurse while providing regular care. All patients were seen on a daily basis by a physician. When delirium was present or suspected a geriatrician was consulted, and the diagnosis was confirmed using the DSM-IV criteria. A delirium was diagnosed if the patient had a Delirium Observational Screening Scale (DOSS) score of ≥3. All types of delirium were included (hypoactive, hyperactive and mixed form). All patients were evaluated for pre- and peri-operative characteristics.

### Predictors of delirium: factors related to frailty

We collected data on main factors related to frailty and subsequently analysed them if prevalence was increased in patients with delirium compared to non-delirious patients.

A standardized history was taken to document comorbidity (cardiac, pulmonary, neurological and renal) of all included patients. Cardiac comorbidity included valve disorders, arrythmia’s, heart failure and ischemic heart disease. Pulmonary comorbidity included chronic obstructive pulmonary disease. Neurological comorbidity included dementia, cerebrovascular accidents, epilepsy or Parkinson's disease. Renal comorbidity included renal impairment defined as a glomerular filtration rate (GFR) of ≤60 ml/min/1.73m^2^. Known predictive factors of postoperative delirium were collected: delirium in the patient’s history, visual and/or hearing impairment, daily alcohol use, smoking, hypertension, hypercholesterolemia and diabetes mellitus. All patients underwent a structured interview on admission assessing these parameters.

The American Society of Anesthesiologist (ASA) status was determined before surgery, from history and physical examination by the attending anesthesiologist.

Functional autonomy was assesed using the basic Activities of Daily Living (ADL) using the Katz-Scale. The inability to complete one or more ADLs was used as cutoff point for physical impairment [15].

Nutritional status was measured using the SNAQ-RC score [16]. A SNAQ-RC Score of 3 or more indicates severe undernourishment.

We were able to prospectively collect the relevant parameters during the study period using a full electronic patient file: Hyperspace Version IU4 (Epic Inc., Verona, Wisconsin, USA) [17]. All collected patient records and information was anonymized and de- identified prior to analysis.

### Predictors of delirium: operative data, hemoglobin and blood transfusion

Anesthesia time was calculated as the duration between tracheal in- and extubation.

Patients underwent surgery for colorectal carcinoma with epidural anaesthesia as a sole technique or as an adjunct to general anaesthesia. All patients who had AAA surgery received general anaesthesia. Patients were treated following the Dutch Society of Anaesthesiologists (NVA) guidelines. Patients had epidural anaesthesia as part of the fast-track protocol [18]. When epidural anesthesia was not eligible (in case of allergies or coagulopathy), as an alternative, a Patient-Controlled analgesia pump (PCA-pump) with Morfine was described.

Hemoglobin (Hb) levels were obtained pre- and post-operatively. Anemia was defined as a Hb <7.6 mmol/L for women and <8.2 mmol/L for men [19]. The amount of peri-operative transfused Packed Cells was listed. Type of surgery was noted for AAA (open repair or endovascular aortic repair (EVAR)) and colorectal cancer (laparotomy or laparoscopic surgery).

### Secondary outcome: consequences of delirium

During follow-up, data on mortality, hospital (surgical ward) stay, and Intensive Care Unit (ICU) stay were registered. Patients having open AAA surgery were admitted to the ICU for at least 24 hours following our hospital protocols. Mortality data were calculated using using the COMPET&T database from the company T&T Eindhoven. Patient destination after hospital discharge was noted (home or new nursing home client). Adverse events were collected during the first 30 postoperative days following the definition as defined by the Association of Surgeons of the Netherlands [20,21].

### Statistics

#### Sample size

Based on previously published studies concerning risk factors of delirium in our hospital, we made an estimation of required sample size [22]. We used the general rule of aiming for 10 events per variable to motivate the sample size.

#### Data analysis

Statistical analysis was performed with SPSS Version 20.0 (SPSS Inc., Chicago, Illonis, USA) software. Univariate analyses (Student t-test and Mann-Whitney U test for continuous data and Chi-square or Fisher exact test or dichotomous data) were performed to evaluate factors that were associated with postoperative delirium.

Pre- and intraoperative parameters that varied significantly (p < 0.05) between delirious and non-delirious patients in the univariate analysis were included in a multivariable analysis. Results with a *P* value < .05 were considered statistically significant.

## Results

A total of 232 patients were included in this study, 51 (22%) having surgery for AAA and 181 (78%) having surgery for colorectal cancer. Postoperative delirium occurred in 35 (15%; 8 after AAA surgery (16%) and 27 after colorectal surgery (15%), *p* = 0.89). Patients having colorectal surgery were older (median 75 years; Interquartile Range (IQR) 10) compared to patients undergoing AAA surgery (median 73 years; IQR 9), *p* = 0.022.

For the 51 AAA patients, 25 (49%) underwent endovascular aneurysm repair (EVAR), and 26 (51%) underwent an open AAA repair using an aortoaortic “straight tube” graft or bifurcated prostheses (Table 1). Delirium was observed in 7 patients after open repair (27%) and in only one patient after EVAR (4%) *p* = 0.050. Patients developed a delirium more frequently after laparotomy (20%) compared to patients having laparoscopic surgery (8%, *p* = 0.024).

**Table 1:** Pre-operative characteristics in patients with AAA or colorectal cancer receiving elective surgery.

	AAA *n* = 51 (%)		Colorectal cancer *n* = 181 (%)		*P* value
**Gender**					
Male	46	(90)	102	(56)	
Female	5	(10)	97	(54)	<0.001
**Age**					
Median age (IQR) ǂ	73	(9)	75	(10)	0.022^b^
Age 65–70 years	17	(33)	39	(22)	0.082
Age 70–79 years	26	(51)	89	(49)	0.819
Age ≥ 80 years	8	(16)	53	(29)	0.051
**Comorbidity**					
Cardiac	23	(45)	53	(29)	0.034
Pulmonary	7	(14)	25	(14)	0.987
Renal impairment	6	(12)	13	(7)	0.383^a^
Neurological	8	(16)	22	(12)	0.507
Diabetes Mellitus	7	(14)	40	(22)	0.216
**Operation**					
EVAR	25	(49)	-	-	
Open procedure	26	(51)	-	-	
Laparoscopy	-	-	83	(46)	
Laparotomy	-	-	98	(54)	
**Delirium**					
Incidence of delirium	8	(16)	27	(15)	0.892

Values in parentheses are percentages unless indicated otherwise; values are ǂ median

(IQR: Interquartile Range)

EVAR: EndoVascular Aortic Repair

P-value is calculated with Chi-square test

a = Fisher exact test

b = Mann-Whitney U test

### Pre-operative factors

Pre-operative factors for delirium were analyzed comparing the delirious patients (*n* = 35) to the non-delirious patients (*n* = 197). The delirious patients were significantly older (median 80 years; IQR 7) compared to the non-delirious patients (median 75 years; IQR 10), *p*<0.001. Among the delirious patients, a delirium in the medical history was reported far more frequently (20%) compared to non-delirious patients (2%; *p* = <0.001).

Physical impairment (Katz-ADL<6) was observed in 29% of the patients who developed a delirium vs. 12% of the patients without a delirium (*p* = 0.012). An ASA score of 3 or higher was more frequently observed in the patients who developed a delirium (66%) vs. the patients who were non-delirious (34%; *p* = <0.001, Table 2).

**Table 2:** Pre-operative variables in relation to onset of postoperative delirium of all. included patients having elective surgery for AAA or colorectal cancer.

	Delirium *n* = 35 (%)		No delirium *n* = 197 (%)		*P* value
**Age**					
Median age (IQR) ǂ	80	(7)	75	(10)	<0.001^b^
**Predictors for delirium**					
Delirium in medical history	7	(20)	3	(2)	<0.001^a^
Daily use of alcohol	9	(26)	60	(30)	0.548
Visual impairment	13	(37)	55	(28)	0.269
Hearing impairment	10	(29)	60	(30)	0.823
Hypertension	23	(66)	95	(48)	0.056
Hypercholesterolemia	13	(37)	68	(35)	0.780
Smoking	6	(17)	31	(16)	0.864
**Physical impairment**					
KATZ-ADL score < 6*	10	(29)	24	(12)	0.012
**Nutritional status**					
SNAQ-RC-score ≥ 3^#^	13	(37)	49	(25)	0.126
**ASA-score ≥ 3**	23	(66)	67	(34)	<0.001
**Living situation**					
Daily nurse visits at home	9	(26)	19	(10)	0.020^a^
Living in nursing home	1	(3)	5	(3)	1.000^a^

Values in parentheses are percentages unless indicated oth

erwise; values are ǂ median (Interquartile Range)

*P*-value is calculated with Chi-square test

a = Fisher exact test

b = Mann-Whitney U test

* Katz-ADL Score 5 or less indicates functional impairment [15]

# SNAQ-RC Score 3 or more indicates severe undernourishment [16]

### Hemoglobin and blood transfusion

Pre-operative hemoglobin levels were lower in the delirious patients (median 7.2 mmol/L; IQR 2.1) compared to the non-delirious patients (median 7.9 mmol/L; IQR 1.7), *p* = 0.025). Data on all variables in relation to onset of postoperative delirium are summarized in Table 3.

**Table 3:** Data on anesthesia, hemoglobin and blood transfusion in relation to onset of postoperative delirium of all included patients having elective surgery.

	Delirium *n* = 35 (%)		No delirium *n* = 197 (%)		*P* value
**Median duration of anesthesia in minutes** (IQR)	131	(74)	117	(75)	0.326^b^
**Perioperative hemoglobin and anemia**					
Median pre-operative Hb in mmol/L (IQR)	7.2	(2.1)	7.9	(1.7)	0.028^b^
Pre-operative anemia* *n* = 232	26	(74)	103	(52)	0.016
Median post-operative Hb in mmol/L (IQR)	6.7	(1.6)	6.8	(1.5)	0.344^b^
Post-operative anemia* *n* = 221	31	(91)	161	(86)	0.584^a^
**Blood transfusion**					
≥ 3 Packed Cells transfused during admission	6	(17)	9	(5)	0.014^a^

Data are presented as n and (%), unless otherwise specified.

IQR = Interquartile Range

*P*-value is calculated with Chi-square test

a = Fishers’ Exact test

b = Mann-Whitney U test

* anemia is defined as a Hb <7.6 mmol/L for women and <8.2 mmol/L for men [19]

### Multivariable analysis

Important risk factors for postoperative delirium were delirium in the medical history (Odds Ratio 12 [95% Confidence Interval 2.7–50]), advancing age (Odds Ratio 2.0 [95% Confidence Interval 1.1–3.8]) per 10 years, and ASA-score ≥3 (Odds Ratio 2.6 [95% Confidence Interval 1.1–5.9], Table 4). The area under the receiver operating characteristic (ROC) curve based on these 3 predictors was 0.76 [95% Confidence Interval 0.66–0.85].

**Table 4:** Univariate and multivariate logistic regression analysis on risk factors for delirium of all included patients having elective surgery for AAA or colorectal cancer.

	OR (95% CI)		Adjusted OR (95% CI)	
Age (+10 years)	2.5	(1.4–4.5)	2.0	(1.1–3.8)
Delirium in medical history	16	(4.0–66)	12	(2.7–50)
Katz-ADL score <6^#^	2.9	(1.2–6.7)	1.7	(0.6–4.4)
ASA score ≥3	3.7	(1.7–7.9)	2.6	(1.1–5.9)
Pre-operative anemia*	2.6	(1.2–5.9)	2.0	(0.8–4.8)

* Anemia is defined as a Hb <7.6 mmol/L for women and <8.2 mmol/L for men[19]

^#^ Katz-ADL Score 5 or less indicates functional impairment [15]

### Outcome after delirium

Several adverse events were more frequently observed in the delirious patients compared to the non-delirious patients. These included pulmonary and cardiac adverse events, renal impairment and urinary retention (Table 5). No differences in surgery related adverse events were observed.

**Table 5:** Adverse events, Hospital length of stay, ICU stay and mortality in relation to onset of postoperative delirium.

	Delirium *n* = 35 (%)		No delirium *n* = 197 (%)		*P* value
**Medical Adverse Events**					
Cardiac	5	(14)	7	(4)	0.021
Pulmonary	9	(26)	12	(6)	0.001
Neurological	2	(6)	1	(1)	0.060
Renal impairment	5	(14)	6	(3)	0.014
Urinary tract infection	3	(9)	5	(3)	0.103
Urinary retention	7	(20)	2	(1)	<0.001
Central venous catheter infection	2	(6)	1	(1)	0.060
**Surgical Adverse Events**					
Wound infection	3	(9)	9	(5)	0.398
Seroma	1	(3)	1	(1)	0.280
Anastomotic leakage	3	(9)	6	(3)	0.139
Re-bleeding requiring intervention	2	(6)	2	(1)	0.109
Ileus	1	(3)	15	(8)	0.478
Superficial wound dehiscence	-	-	1	(1)	1.000
Complete wound dehiscence	-	-	2	(1)	1.000
Intra-abdominal abscess	2	(6)	3	(2)	0.165
Embolectomy	-	-	1	(1)	1.000
Other complication^#^	1	(4)	8	(4)	1.000
**Length of stay**					
Median total hospital length of stay in days (IQR)	12	(12)	7	(5)	<0.001^b^
Admission to ICU	17	(49)	32	(16)	<0.001^a^
ICU stay in days ≥ 2	13	(37)	14	(7)	<0.001
**Mortality**					
30-day mortality	3	(9)	1	(1)	0.011
6-month mortality	7	(20)	6	(3)	0.001
**Discharge destination**					
New nursing home client after discharge	5/32	(16)	15/196	(8)	0.171
Discharge same living situation	27/32	(84)	181/196	(92)	0.337

Data are presented as n and (%), unless otherwise specified.

*P*-value is calculated with Fisher's exact test

a = Chi-square test

b = Mann-Whitney U test

# other complications: intoxication with morphine requiring ICU admission, bleeding gastric stress ulcer, iatrogenic injury (spleen, bladder and gallbladder), mild ischemic colitis after AAA surgery.

The total length of hospital stay was higher (median 12 days; IQR 12) in the delirious patients vs. the non-delirious patients (median 7 days; IQR 5), *p* = <0.001.

Intensive Care Unit (ICU) stay for 2 days or longer was observed more frequently in patients with a delirium (49%) compared to patients without a delirium (16%, *p* = <0.001). Occurrence of delirium was related to an increase in length of hospital stay (plus 7.7 days) and ICU stay (plus 2.1 days), after adjustment for age, delirium in medical history, Katz-ADL score, ASA score and pre-operative anemia.

Thirty-day mortality was significantly higher (9%) in the delirious patients compared to the non-delirious patients (1%, *p* = 0.011, Table 5). This difference remained statistically significant in a logistic regression model that corrected for age, ASA score and previous delirium.

**Fig 1:**
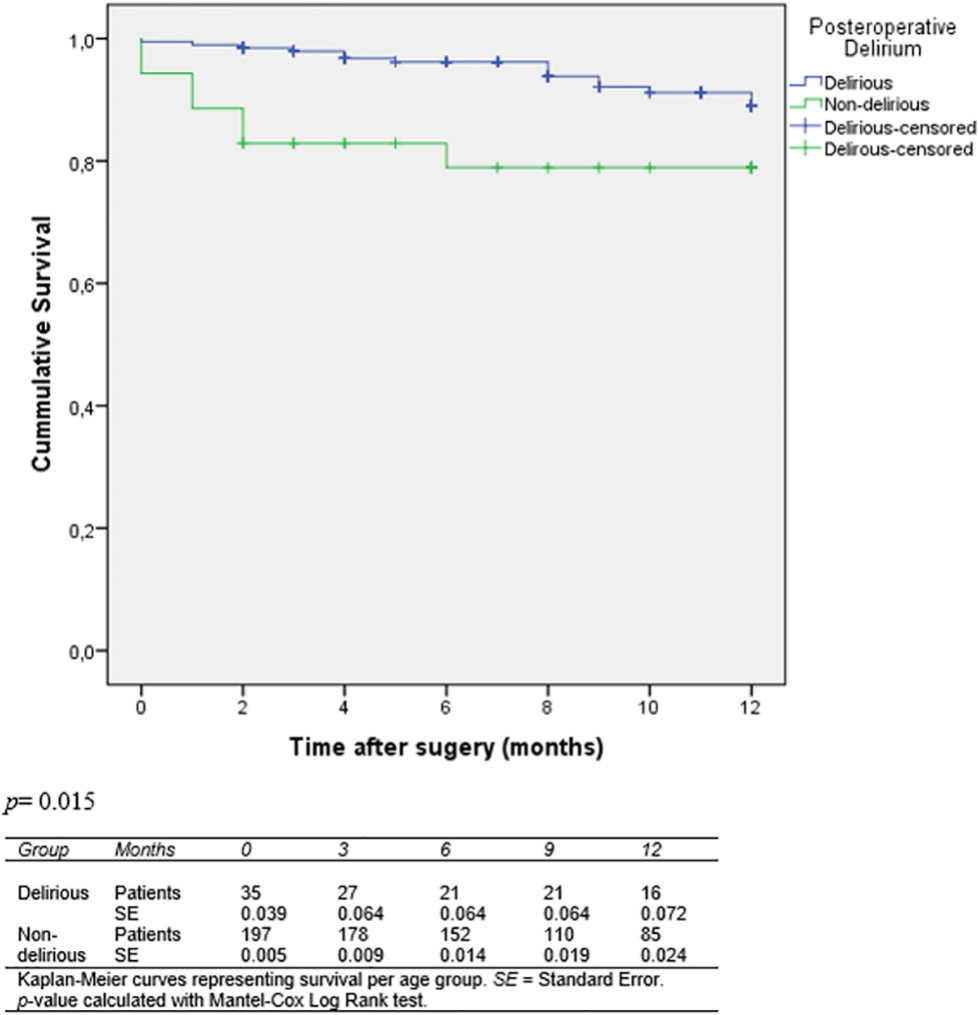
Survival curve for delirious and non-delirious patients having elective AAA or colorectal surgery.

Patients with a delirium had a significantly higher mortality compared to the non-delirious patients (*p* = 0.015, Fig 1).

## Discussion

The number of elderly patients undergoing elective major abdominal surgery for AAA or colorectal cancer is expected to increase. As high age is a main risk factor for delirium, identification of patients at risk for delirium is of major importance. Delirium is related to an increase in morbidity, mortality, length of stay and care home placement [23]. Most importantly, delirium could be prevented in approximately 30–40% of the cases [24,25]. Research concerning postoperative delirium is increasing. However, large reliable studies on predictors of postoperative delirium are rare and most studies focus on cardiac- or orthopedic surgery [26].

Our reported incidence rate of post-operative delirium (15%) is comparable with other studies in recent literature (11–18%) [12,27–29]. Patients developed a delirium more often after open repair (27%) compared to EVAR (4%). Despite the minimal invasive nature of the EVAR technique, previous RCT studies reported similar results of EVAR and open repair for elective AAA surgery [30,31]. Interestingly, the delirium-related costs were not included in previous cost-analysis reporting EVAR is not cost-effective compared with open repair [32]. For elderly patients at risk for delirium, EVAR could be preferable to open surgery.

Both minimal invasive surgical techniques (EVAR and laparoscopic surgery) were associated with lower rates of post-operative delirium. Probably, this could be explained by selection bias. Patients with or colorectal cancer in a more advanced stage may have been more likely to undergo open surgery. In addition, more extensive surgery may have contributed to increased postoperative pain and use of narcotics.

As delirium was more frequently observed after open surgery, decisions regarding surgical approach (laparoscopic or open surgery) for the treatment of colon cancer should be taken into consideration, especially in elderly patients at risk for delirium.

A previous delirium, advancing age, and ASA-score were identified as independent risk factors for delirium. However, it is important to keep in mind that delirium in the patients’ medical history was not highly prevalent in this study population. The identified predictive factors are also reported in earlier studies, but are largely based on non-abdominal surgery patient populations [33–35]. Prevalence of delirium increases with age. At multivariable analysis, we identified age as an independent predictor of delirium, consistent with literature [10]. This may explain why factors describing frailty, such as physical impairment, nutritional status, living arrangement, visual and hearing impairment, were not significant after multivariable correction.

We found that 31% (19/61) of the octogenarians developed a postoperative delirium. This is of major importance, as population projections indicate that the number of octogenarians will double during the next 30 years [36]. Reports on incidence rates of delirium after emergency major surgery are higher (18–33%), but are not uniform. This may be explained by the large heterogeneity of included populations and the use of different diagnostic tools and procedures [37–39]. Many non-pharmalogical preventive interventions, for example help in patient orientation, care for auditive and visual impairment, ensuring the day-sleep cycle, prevention of dehydration and malnutrition, have proved highly effective in an elective setting but are not easily translatable to an emergency setting [40]. In addition, timely direct treatment or optimization of identified predictive factors could potentially help in reducing delirium. However, most factors are not suitable for optimization because they are irreversible (i.e. age, ASA-score, delirium in patients’ history). Still, preventive strategies should be initiated in these patients identified as at risk for delirium. In this study this included preventive nursing actions such as help in patients orientation, care for auditory and visual impairment, prevention of dehydration and malnutrition. When necessary, low dose prophylactic anti-psychotic (Haloperidol) was prescribed based on judgement of the consulting geriatrician.

Another interesting observation in our study is the role of pre-operative hemoglobin and delirium. *Böhner et al*, did not find that pre-operative hemoglobin was linked to postoperative delirium after vascular surgery [26]. In contrast, *Joostens et al* found that in men, decreased pre-operative hemoglobin levels increased the risk of postoperative delirium in geriatric patients [41]. In our study, hemoglobin levels were not predictive of delirium after multivariable analysis with correction for confounders.

In case of elective major surgery, pre-operative treatment to increase hemoglobin levels might possibly prevent occurrence of postoperative delirium. Further research is required to explore this.

Delirium was related to multiple adverse events and increase in hospital stay. The question of whether delirium is a symptom of other postoperative complications or whether a delirium increases the risk of postoperative adverse events remains to be answered [42]. For instance, delirium could be a complication of an acquired pneumonia. On the other hand, pneumonia could be a result of aspiration provoked by delirium. In this study it remains unknown if the increased incidence of other adverse events are a cause or an effect of postoperative delirium. Probably, reducing occurrence of postoperative delirium could reduce the associated adverse outcomes and subsequently reduce costs.

### Limitations

In this study we used a DOSS-score of ≥3 as highly indicative for delirium. Despite the use of a validated instrument, this may be an imperfect reflection of delirium. Our reported results could be an underestimation of delirium incidence by missing clinical subtypes such as the hypoactive delirium. Patients were not screened for psychiatric disease by admission. This is a limitation since earlier studies identified depression as an idepentent risk factor for delirium [43].

We included AAA and colorectal surgery patients and performed a pooled multivariable analysis. This may have led to a less correct identification of possible risk factors for delirium per disease group. However, incidence rates of post-operative delirium were comparable in both groups.

Medication, data regarding the anesthesia protocol and perioperative pain therapy was not included as possible predictor for delirium in this study. However, several earlier studies confirmed medication as a risk factor for postoperative delirium and therefore this should be included in further research. This is of great importance, because the increase of the elderly population, also the number of frail elderly with multimorbidity and polypharmacy will increase in future. In addition, medication could be an easy modifiable risk factor.

Another limitation of this study is the restriction of data collection to one hospital, although we consider this center representative for European large non-academic hospitals.

Finally, the number of patients in the present study does not permit strong conclusions, particularly since the numbers of patients with a delirium were relatively small. A prediction model for delirium in elderly having major elective surgery should be developed in larger series, with attention to validation and updating of existing prediction models [44].

## Conclusions

A delirium in the medical history, advanced age, and ASA-score may assist in defining patients at increased risk for delirium. Postoperative delirium is a frequent complication among elderly patients. Since occurrence of delirium is related to an increase in adverse events, length of hospital stay and mortality, further attention for prevention of delirium is essential in elderly patients undergoing major surgery.

## Supporting Information

S1 File Data are available on request from the institutional review board (Advisory commission involving Human Subjects Research Amphia Hospital-AMOA) of the Amphia hospital. (PDF)

## References

[1] Bekker AY, Weeks EJ (2003) Cognitive function after anaesthesia in the elderly. Best Pract Res Clin Anaesthesiol 17: 259–272. 12817919 10.1016/s1521-6896(03)00005-3

[2] Bilotta F, Doronzio A, Stazi E, Titi L, Zeppa IO, Cianchi A, et al (2011) Early postoperative cognitive dysfunction and postoperative delirium after anaesthesia with various hypnotics: study protocol for a randomised controlled trial—the PINOCCHIO trial. Trials 12: 170 10.1186/1745-6215-12-170 21733178 PMC3155116

[3] Deiner S, Silverstein JH (2009) Postoperative delirium and cognitive dysfunction. Br J Anaesth 103 Suppl 1: i41–46. 10.1093/bja/aep291 20007989 PMC2791855

[4] Laurila JV, Laakkonen ML, Tilvis RS, Pitkala KH (2008) Predisposing and precipitating factors for delirium in a frail geriatric population. J Psychosom Res 65: 249–254. 10.1016/j.jpsychores.2008.05.026 18707947

[5] Rudolph JL, Marcantonio ER, Culley DJ, Silverstein JH, Rasmussen LS, Crosby GJ, et al (2008) Delirium is associated with early postoperative cognitive dysfunction. Anaesthesia 63: 941–947. 10.1111/j.1365-2044.2008.05523.x 18547292 PMC2562627

[6] Steinmetz J, Rasmussen LS (2010) The elderly and general anesthesia. Minerva Anestesiol 76: 745–752. 20820153

[7] Freter SH, Dunbar MJ, MacLeod H, Morrison M, MacKnight C, Rockwood K (2005) Predicting post-operative delirium in elective orthopaedic patients: the Delirium Elderly At-Risk (DEAR) instrument. Age Ageing 34: 169–171. 15713861 10.1093/ageing/afh245

[8] Kalisvaart KJ, Vreeswijk R, de Jonghe JF, van der Ploeg T, van Gool WA, Eikelenboom P (2006) Risk factors and prediction of postoperative delirium in elderly hip-surgery patients: implementation and validation of a medical risk factor model. J Am Geriatr Soc 54: 817–822. 16696749 10.1111/j.1532-5415.2006.00704.x

[9] Marcantonio ER, Goldman L, Mangione CM, Ludwig LE, Muraca B, Haslauer CM, et al (1994) A clinical prediction rule for delirium after elective noncardiac surgery. JAMA 271: 134–139. 8264068

[10] Noimark D (2009) Predicting the onset of delirium in the post-operative patient. Age Ageing 38: 368–373. 10.1093/ageing/afp024 19297372

[11] Clegg A, Young J, Iliffe S, Rikkert MO, Rockwood K (2013) Frailty in elderly people. Lancet 381: 752–762. 10.1016/S0140-6736(12)62167-9 23395245 PMC4098658

[12] Korc-Grodzicki B, Sun SW, Zhou Q, Iasonos A, Lu B, Root JC, et al (2014) Geriatric Assessment as a Predictor of Delirium and Other Outcomes in Elderly Patients With Cancer. Ann Surg. 10.1097/SLA.0000000000000742 PMC4837653 24887981

[13] Schuurmans MJ, Shortridge-Baggett LM, Duursma SA (2003) The Delirium Observation Screening Scale: a screening instrument for delirium. Res Theory Nurs Pract 17: 31–50. 12751884 10.1891/rtnp.17.1.31.53169

[14] Scheffer AC, van Munster BC, Schuurmans MJ, de Rooij SE (2011) Assessing severity of delirium by the Delirium Observation Screening Scale. Int J Geriatr Psychiatry 26: 284–291. 10.1002/gps.2526 20665557

[15] Wallace M, Shelkey M, Hartford Institute for Geriatric N (2007) Katz Index of Independence in Activities of Daily Living (ADL). Urol Nurs 27: 93–94. 17390935

[16] Kruizenga HM, Seidell JC, de Vet HC, Wierdsma NJ, van Bokhorst-de van der Schueren MA (2005) Development and validation of a hospital screening tool for malnutrition: the short nutritional assessment questionnaire (SNAQ). Clin Nutr 24: 75–82. 15681104 10.1016/j.clnu.2004.07.015

[17] (2012) Epic© Hyperspace©. IU4 ed. Verona, Wilconsin, United States.

[18] Kehlet H, Wilmore DW (2005) Fast-track surgery. Br J Surg 92: 3–4. 15635603 10.1002/bjs.4841

[19] Desormais I, Aboyans V, Bura A, Constans J, Cambou JP, Messas E, et al (2014) Anemia, an independent predictive factor for amputation and mortality in patients hospitalized for peripheral artery disease. Eur J Vasc Endovasc Surg 48: 202–207. 10.1016/j.ejvs.2014.04.005 24935912

[20] Marang-van de Mheen PJ, Stadlander MC, Kievit J (2006) Adverse outcomes in surgical patients: implementation of a nationwide reporting system. Qual Saf Health Care 15: 320–324. 17074866 10.1136/qshc.2005.016220 PMC2565813

[21] Marang-van de Mheen PJ, van Duijn-Bakker N, Kievit J (2007) Surgical adverse outcomes and patients' evaluation of quality of care: inherent risk or reduced quality of care? Qual Saf Health Care 16: 428–433. 18055886 10.1136/qshc.2006.021071 PMC2653187

[22] Raats JW, Steunenberg SL, Crolla RM, Wijsman JH, Te Slaa A, van der Laan L (2015) Postoperative delirium in elderly after elective and acute colorectal surgery: A prospective cohort study. Int J Surg 18: 216–219. 10.1016/j.ijsu.2015.04.080 25937152

[23] Kiran RP, Attaluri V, Hammel J, Church J (2013) A novel nomogram accurately quantifies the risk of mortality in elderly patients undergoing colorectal surgery. Ann Surg 257: 905–908. 10.1097/SLA.0b013e318269d337 23001078

[24] Inouye SK, Bogardus ST Jr., Charpentier PA, Leo-Summers L, Acampora D, Holford TR, et al (1999) A multicomponent intervention to prevent delirium in hospitalized older patients. N Engl J Med 340: 669–676. 10053175 10.1056/NEJM199903043400901

[25] Marcantonio ER, Flacker JM, Wright RJ, Resnick NM (2001) Reducing delirium after hip fracture: a randomized trial. J Am Geriatr Soc 49: 516–522. 11380742 10.1046/j.1532-5415.2001.49108.x

[26] Bohner H, Hummel TC, Habel U, Miller C, Reinbott S, Yang Q, et al (2003) Predicting delirium after vascular surgery: a model based on pre- and intraoperative data. Ann Surg 238: 149–156. 12832977 10.1097/01.sla.0000077920.38307.5f PMC1422662

[27] Tei M, Ikeda M, Haraguchi N, Takemasa I, Mizushima T, Ishii H, et al (2010) Risk factors for postoperative delirium in elderly patients with colorectal cancer. Surg Endosc 24: 2135–2139. 10.1007/s00464-010-0911-7 20177939

[28] Patti R, Saitta M, Cusumano G, Termine G, Di Vita G (2011) Risk factors for postoperative delirium after colorectal surgery for carcinoma. Eur J Oncol Nurs 15: 519–523. 10.1016/j.ejon.2011.01.004 21333597

[29] Koebrugge B, van Wensen RJ, Bosscha K, Dautzenberg PL, Koning OH (2010) Delirium after emergency/elective open and endovascular aortoiliac surgery at a surgical ward with a high-standard delirium care protocol. Vascular 18: 279–287. 20822723 10.2310/6670.2010.00052

[30] De Bruin JL, Baas AF, Buth J, Prinssen M, Verhoeven EL, Cuypers PW, et al (2010) Long-term outcome of open or endovascular repair of abdominal aortic aneurysm. N Engl J Med 362: 1881–1889. 10.1056/NEJMoa0909499 20484396

[31] United Kingdom ETI, Greenhalgh RM, Brown LC, Powell JT, Thompson SG, Epstein D, et al (2010) Endovascular versus open repair of abdominal aortic aneurysm. N Engl J Med 362: 1863–1871. 10.1056/NEJMoa0909305 20382983

[32] Epstein D, Sculpher MJ, Powell JT, Thompson SG, Brown LC, Greenhalgh RM (2014) Long-term cost-effectiveness analysis of endovascular versus open repair for abdominal aortic aneurysm based on four randomized clinical trials. Br J Surg 101: 623–631. 10.1002/bjs.9464 24664537

[33] Weed HG, Lutman CV, Young DC, Schuller DE (1995) Preoperative identification of patients at risk for delirium after major head and neck cancer surgery. Laryngoscope 105: 1066–1068. 7564837 10.1288/00005537-199510000-00011

[34] Zakriya KJ, Christmas C, Wenz JF Sr., Franckowiak S, Anderson R, Sieber FE (2002) Preoperative factors associated with postoperative change in confusion assessment method score in hip fracture patients. Anesth Analg 94: 1628–1632, table of contents. 12032042 10.1097/00000539-200206000-00050

[35] Litaker D, Locala J, Franco K, Bronson DL, Tannous Z (2001) Preoperative risk factors for postoperative delirium. Gen Hosp Psychiatry 23: 84–89. 11313076 10.1016/s0163-8343(01)00117-7

[36] Nationaal Kompas.

[37] Ansaloni L, Catena F, Chattat R, Fortuna D, Franceschi C, Mascitti P, et al (2010) Risk factors and incidence of postoperative delirium in elderly patients after elective and emergency surgery. Br J Surg 97: 273–280. 10.1002/bjs.6843 20069607

[38] Engelberger S, Zurcher M, Schuld J, Viehl CT, Kettelhack C (2012) Postoperative course after emergency colorectal surgery for secondary peritonitis in the elderly is often complicated by delirium. Int Surg 97: 129–134. 10.9738/CC125.1 23102078 PMC3723209

[39] Raats JW SS, Crolla RMPH, Wijsman JH, te Slaa A, van der Laan L (2015) Postoperative delirium in elderly patients after elective and emergency colorectal surgery Journal of Gastrointestinal Surgery. 10.1016/j.ijsu.2015.04.080 25937152

[40] Siddiqi N, Stockdale R, Britton AM, Holmes J (2007) Interventions for preventing delirium in hospitalised patients. Cochrane Database Syst Rev: CD005563 17443600 10.1002/14651858.CD005563.pub2

[41] Joosten E, Lemiengre J, Nelis T, Verbeke G, Milisen K (2006) Is anaemia a risk factor for delirium in an acute geriatric population? Gerontology 52: 382–385. 16914932 10.1159/000095126

[42] Olin K, Eriksdotter-Jonhagen M, Jansson A, Herrington MK, Kristiansson M, Permert J (2005) Postoperative delirium in elderly patients after major abdominal surgery. Br J Surg 92: 1559–1564. 16231283 10.1002/bjs.5053

[43] Schneider F, Bohner H, Habel U, Salloum JB, Stierstorfer A, Hummel TC, et al (2002) Risk factors for postoperative delirium in vascular surgery. Gen Hosp Psychiatry 24: 28–34. 11814531 10.1016/s0163-8343(01)00168-2

[44] Steyerberg EW (2009) Clinical Prediction Models; Gail M, editor: Springer Science+Business Media.

