# Supplementary material for: Risk Factors and Outcomes for Postoperative Delirium after Major Surgery in Elderly Patients
Source: PLoS One. 2015 Aug 20;10(8):e0136071. doi: 10.1371/journal.pone.0136071 (PMC4546338; doi:10.1371/journal.pone.0136071)
Supplement: S1 File — (PDF) [file pone.0136071.s001.pdf]

Table 1 Pre-operative characteristics in patients with AAA or colorectal cancer receiving elective surgery

	AAA	Colorectal cancer	*P* value
	n= 51 (%)	*n*= 181 (%)	
**Gender**			
Male	46 (90)	102 (56)	
Female	5 (10)	97 (54)	<0.001
**Age**			
Median age (IQR) □	73 (9)	75 (10)	0.022[b]
Age 65-70 years	17 (33)	39 (22)	0.082
Age 70-79 years	26 (51)	89 (49)	0.819
Age ≥ 80 years	8 (16)	53 (29)	0.051
**Comorbidity**			
Cardiac	23 (45)	53 (29)	0.034
Pulmonary	7 (14)	25 (14)	0.987
Renal impairment	6 (12)	13 (7)	0.383[a]
Neurological	8 (16)	22 (12)	0.507
Diabetes Mellitus	7 (14)	40 (22)	0.216
**Operation**			
EVAR	25 (49)	-	
Open procedure	26 (51)	-	
Laparoscopy	-	83 (46)	
Laparotomy	-	98 (54)	
**Delirium**			
Incidence of delirium	8 (16)	27 (15)	0.892

Values in parentheses are percentages unless indicated otherwise; values are □ median (IQR: Interquartile Range)
EVAR: EndoVascular Aortic Repair
P-value is calculated with Chi-square test
a= Fisher exact test
b= Mann-Whitney U test

Table 2 Pre-operative variables in relation to onset of postoperative delirium of all included patients having elective surgery for AAA or colorectal cancer

	Delirium<br>*n*= 35 (%)	No delirium<br>*n*= 197 (%)	*P* value
**Age**			
Median age (IQR) □	80 (7)	75 (10)	<0.001[b]
**Predictors for delirium**			
Delirium in medical history	7 (20)	3 (2)	<0.001[a]
Daily use of alcohol	9 (26)	60 (30)	0.548
Visual impairment	13 (37)	55 (28)	0.269
Hearing impairment	10 (29)	60 (30)	0.823
Hypertension	23 (66)	95 (48)	0.056
Hypercholesterolemia	13 (37)	68 (35)	0.780
Smoking	6 (17)	31 (16)	0.864
**Physical impairment**			
KATZ-ADL score < 6*	10 (29)	24 (12)	0.012
**Nutritional status**			
SNAQ-RC-score ≥ 3#	13 (37)	49 (25)	0.126
**ASA-score ≥ 3**	23 (66)	67 (34)	<0.001
**Living situation**			
Daily nurse visits at home	9 (26)	19 (10)	0.020[a]
Living in nursing home	1 (3)	5 (3)	1.000[a]

Values in parentheses are percentages unless indicated otherwise; values are □ median (Interquartile Range)
P-value is calculated with Chi-square test
a= Fisher exact test
b= Mann-Whitney U test
* Katz-ADL Score 5 or less indicates functional impairment [42]
# SNAQ-RC Score 3 or more indicates severe undernourishment [16]

Table 3 Data on anesthesia, hemoglobin and blood transfusion in relation to onset of postoperative delirium of all included patients having elective surgery

	Delirium *n*= 35 (%)		No delirium *n*= 197 (%)		*P* value
**Median duration of anesthesia in minutes** (IQR)	131	(74)	117	(75)	0.326[b]
**Perioperative hemoglobin and anemia**					
Median pre-operative Hb in mmol/L (IQR)	7.2	(2.1)	7.9	(1.7)	0.028[b]
Pre-operative anemia* *n*=232	26	(74)	103	(52)	0.016
Median post-operative Hb in mmol/L (IQR)	6.7	(1.6)	6.8	(1.5)	0.344[b]
Post-operative anemia* *n*=221	31	(91)	161	(86)	0.584[a]
**Blood transfusion**					
≥ 3 Packed Cells transfused during admission	6	(17)	9	(5)	0.014[a]

Data are presented as n and (%), unless otherwise specified.
IQR = Interquartile Range
P-value is calculated with Chi-square test
a= Fishers' Exact test
b= Mann-Whitney U test
* anemia is defined as a Hb <7.6 mmol/L for women and <8.2 mmol/L for men [18]

Table 4 Univariate and multivariate logistic regression analysis on risk factors for delirium of all included patients having elective surgery for AAA or colorectal cancer

	OR	(95% CI)	Adjusted OR	(95% CI)
Age (+10 years)	2.5	(1.4-4.5)	2.0	(1.1-3.8)
Delirium in medical history	16	(4.0-66)	12	(2.7-50)
Katz-ADL score <6#	2.9	(1.2-6.7)	1.7	(0.6-4.4)
ASA score ≥3	3.7	(1.7-7.9)	2.6	(1.1-5.9)
Pre-operative anemia*	2.6	(1.2-5.9)	2.0	(0.8-4.8)

* Anemia is defined as a Hb <7.6 mmol/L for women and <8.2 mmol/L for men[18]

Katz-ADL Score 5 or less indicates functional impairment [42]

Table 5 Adverse events, Hospital length of stay, ICU stay and mortality in relation to onset of postoperative delirium

	Delirium		No delirium		*P* value
	n= 35 (%)		*n*= 197 (%)		
**Medical Adverse Events**					
Cardiac	5	(14)	7	(4)	0.021
Pulmonary	9	(26)	12	(6)	0.001
Neurological	2	(6)	1	(1)	0.060
Renal impairment	5	(14)	6	(3)	0.014
Urinary tract infection	3	(9)	5	(3)	0.103
Urinary retention	7	(20)	2	(1)	<0.001
Central venous catheter infection	2	(6)	1	(1)	0.060
**Surgical Adverse Events**					
Wound infection	3	(9)	9	(5)	0.398
Seroma	1	(3)	1	(1)	0.280
Anastomotic leakage	3	(9)	6	(3)	0.139
Re-bleeding requiring intervention	2	(6)	2	(1)	0.109
Ileus	1	(3)	15	(8)	0.478
Superficial wound dehiscence	-	-	1	(1)	1.000
Complete wound dehiscence	-	-	2	(1)	1.000
Intra-abdominal abscess	2	(6)	3	(2)	0.165
Embolectomy	-	-	1	(1)	1.000
Other complication[#]	1	(4)	8	(4)	1.000
**Length of stay**					
Median total hospital length of stay in days (IQR)	12	(12)	7	(5)	<0.001[b]
Admission to ICU	17	(49)	32	(16)	<0.001[a]
ICU stay in days ≥ 2	13	(37)	14	(7)	<0.001
**Mortality**					
30-day mortality	3	(9)	1	(1)	0.011
6-month mortality	7	(20)	6	(3)	0.001
**Discharge destination**					
New nursing home client after discharge	5/32	(16)	15/196	(8)	0.171
Discharge same living situation	27/32	(84)	181/196	(92)	0.337

Data are presented as n and (%), unless otherwise specified.
P-value is calculated with Fisher's exact test
a=Chi-square test
b= Mann-Whitney U test
# other complications: intoxication with morphine requiring ICU admission, bleeding gastric stress ulcer, iatrogenic injury (spleen, bladder and gallbladder), mild ischemic colitis after AAA surgery.

Figure 1 Survival curve for delirious and non-delirious patients having elective AAA or colorectal surgery

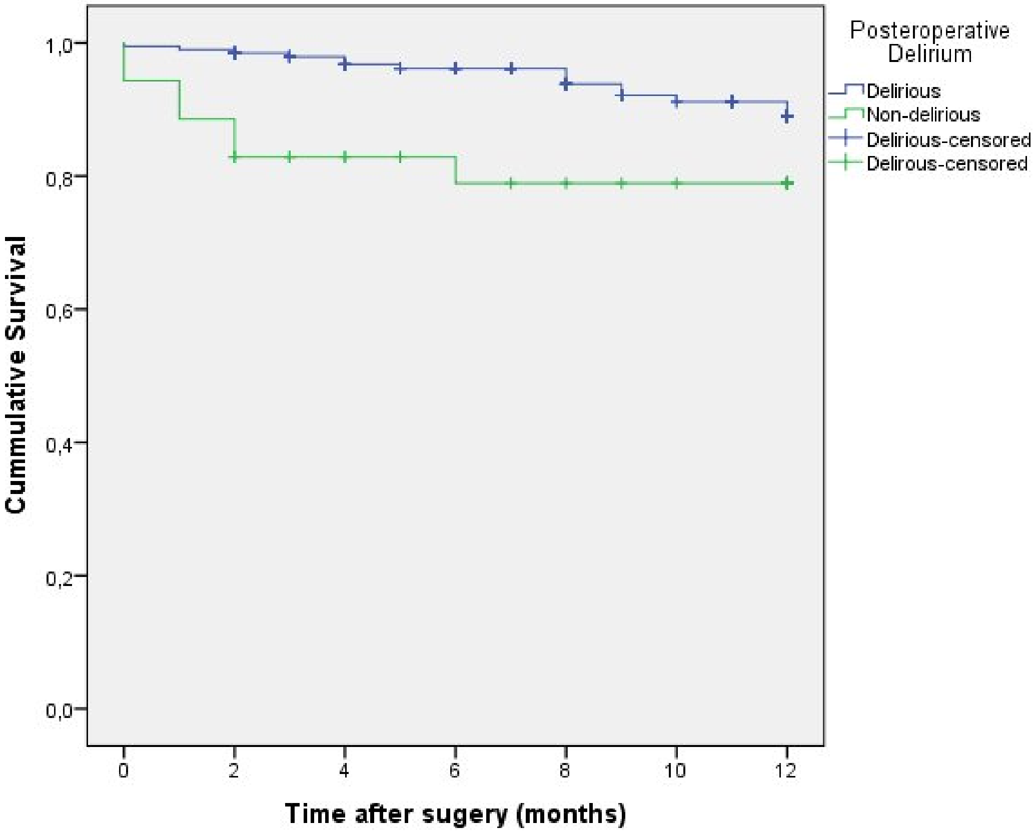

p= 0.015

Group	*Months*	*0*	*3*	*6*	*9*	*12*
Delirious	Patients	35	27	21	21	16
	SE	0.039	0.064	0.064	0.064	0.072
Non-delirious	Patients	197	178	152	110	85
	SE	0.005	0.009	0.014	0.019	0.024

Kaplan-Meier curves representing survival per age group. *SE* = Standard Error.
p-value calculated with Mantel-Cox Log Rank test.
